# A data-driven reduced-order surrogate model for entire elastoplastic simulations applied to representative volume elements

**DOI:** 10.1038/s41598-023-38104-x

**Published:** 2023-08-07

**Authors:** S. Vijayaraghavan, L. Wu, L. Noels, S. P. A. Bordas, S. Natarajan, L. A. A. Beex

**Affiliations:** 1 Faculty of Science, Technology and Medicine, University of Luxembourg, 6 Avenue de la Fonte, Esch-Sur-Alzette, Luxembourg; 2 University of Liege, Bât. B52/3 Computational & Multiscale Mechanics of Materials, Quartier Polytech 1, allée de la Découverte 9, 4000 Liège, Belgium; 3 Department of Mechanical Engineering, Indian Institute of Technology, Madras, Chennai, 600036 India

**Keywords:** Aerospace engineering, Civil engineering, Mechanical engineering, Computational methods, Computational science

## Abstract

This contribution discusses surrogate models that emulate the solution field(s) in the entire simulation domain. The surrogate uses the most characteristic modes of the solution field(s), in combination with neural networks to emulate the coefficients of each mode. This type of surrogate is well known to rapidly emulate flow simulations, but rather new for simulations of elastoplastic solids. The surrogate avoids the iterative process of constructing and solving the linearized governing equations of rate-independent elastoplasticity, as necessary for direct numerical simulations or (hyper-)reduced-order-models. Instead, the new plastic variables are computed only once per increment, resulting in substantial time savings. The surrogate uses a recurrent neural network to treat the path dependency of rate-independent elastoplasticity within the neural network itself. Because only a few of these surrogates have been developed for elastoplastic simulations, their potential and limitations are not yet well studied. The aim of this contribution is to shed more light on their numerical capabilities in the context of elastoplasticity. Although more widely applicable, the investigation focuses on a representative volume element, because these surrogates have the ability to both emulate the macroscale stress-deformation relation (which drives the multiscale simulation), as well as to recover all microstructural quantities within each representative volume element.

## Introduction

Rate-independent elastoplasticity is ‘path-dependent’, i.e. ‘history-dependent’. This means that the current stress state does not only depend on the current deformation, but also on ‘all previous deformations’—i.e. the ‘history’, the ‘path’, or the ‘trajectory’ of the deformation. The specification ‘rate-independent’ entails that the path of the deformation is of influence, but not at which rate the (load) path was taken.

Rate-independent elastoplasticity uses plastic variables to quantify its history (i.e. ‘history variables’, ‘process variables’ or ‘internal (state) variables’). The number of plastic variables depends on the constitutive model and the number of spatial dimensions considered, but not on the complexity of the history. In other words, the number of plastic variables does not increase as the deformation history complexifies.

Most recent studies that investigate and develop neural networks (NNs) to rapidly emulate rate-independent elastoplastic behaviours focus on *recurrent* NNs, instead of *feed-forward* neural networks^1^. The reason is that *recurrent* NNs (RNNs) not only emulate the quantity of current interest, they also emulate auxiliary quantities, whose sole purpose is to quantify the RNN’s history. These auxiliary quantities are used by the RNN to affect its *next* prediction (i.e. the prediction for the next time increment). Hence, the auxiliary quantities, called ‘hidden states’ or ‘hidden variables’ in RNN terminology, play the same role for the RNN as the plastic variables for elastoplasticity. Similar to the number of plastic variables elastoplasticity, the number of hidden states does not increase as the RNN’s history grows.

Although the previous paragraph may seem to argue against the use of *feed-forward* NNs (FNNs) for elastoplasticity, this is not intended. FNNs, as investigated for elastoplasticity in pioneering studies^2–5^ but also recently^6^, are for instance useful if the entire load path is known in advance. In this case, the input of the FNN is the entire load path and its output is the quantity of interest for the entire load path (i.e. for all time increments at once). FNNs can also be used for each time step independently, if the load path is (close to) monotonically increasing.

Often however, the load path is not known in advance and/or the load path is more complex than monotonically increasing. Alternatively, Huang et al.^7^ have included the path-dependence of elastoplasticity in an FNN by incorporating the entire load path up to the current time increment as input. In that way, the FNN’s output remains limited to the current time step.

Currently, RNNs seem more popular than FNNs for the emulation of elastoplastic behaviours. Recent investigations of RNNs for elastoplasticity may be classified in different branches.

### RNNs for elastoplastic constitutive responses

The main branch^8–16^ investigates RNNs to emulate the computations of new stress states, as well as the sensitivity of the new stress state to the deformation (i.e. the so-called consistent tangent moduli). This accelerates mechanical simulations by rapidly emulating the construction of the linearized governing equations. It also provides a large degree of flexibility (e.g. different geometries, different meshes, and different boundary conditions can be considered).

A major application of interest to this branch is the data-driven discovery of macroscopic stress-deformation relations governed by representative volume elements (RVE) computations, in order to rapidly emulate time-consuming multiscale simulations^1,8,9,14^.

It is worth noting that several of these works do not simply exploit RNNs, but incorporate knowledge of elastoplastic constitutive theory in order to improve their accuracy^12,13^.

### RNN-POD surrogates for entire RVEs

Another (smaller) branch aims to rapidly emulate the entire displacement field of an elastoplastic simulation (for each time increment of the simulation). The only issue that remains to be computed in such emulators is the field of new plastic variables (once per increment). The primary and quintessential advantage over the approach of the previous paragraphs (when applied to an RVE) is that all microstructural quantities remain available.

The disadvantage is a reduced flexibility, because the geometry and the mesh must remain the same. Hence, the RNN is generally restricted to only treat variations of the mechanical parameters and variations of the load path (e.g. the path of the macroscale deformation in case of RVEs).

Wu and Noels investigated a similar approach^17^. Their framework regards a wide variety of loading states for which three variables are required to parametrize the currently imposed loading on a 2D elastoplastic RVE (i.e. three variables are necessary to quantify the 2D, invariant macroscale deformation imposed on a 2D RVE). The elastoplastic constitutive model furthermore accounts for large deformations and rotations, which also occur in their numerical tests.

The issue with the study of Wu and Noels^17^ is that the quantity of interest is not the primary solution field (i.e. the displacement field), but the fields of the plastic multiplier and the von Mises stress. The advantage is that these fields are indeed available in an instant, but the disadvantage for true multiscale simulations is that the macroscale stress-deformation response (that drives the simulation) is not available. Thus, the framework^17^ can only be used in combination with another RNN that emulates the macroscale stress-deformation relation (which was indeed published two years prior^9^).

A framework that emulates the displacement field once per increment, as is the focus of the current contribution, provides the flexibility to both emulate the macroscale stress-deformation relation that drives the multiscale simulation, as well as any microstructural quantity inside each RVE.

However, if the RNN’s output is the entire displacement field (for each increment), the number of output variables is substantial; the RNN must emulate the displacement vector of each finite element node (besides the hidden states). This yields complex RNNs, with long training times.

In order to keep an RNN’s architecture relatively simple, its number of outputs may be restricted. This can be achieved by first calculating a number of (orthonormal) displacements fields (i.e. ‘modes’) that are most characteristic for the displacement fields that were previously predicted by some training simulations. The RNN then only has to emulate the coefficient of each mode.

These types of surrogates (also using FNNs instead of RNNs) have been developed for a wide variety of engineering simulations^18–23^, but only three were developed for elastoplastic simulations^24–26^, to the best of the authors’ knowledge.

The modes (which together form a basis) are obtained by first performing numerous direct numerical simulations (DNS), and storing the predicted displacement fields. One then applies singular value decomposition (SVD) to these displacement fields, in order to retrieve the modes that are most characteristic for all displacement fields. In model-order-reduction terminology, this approach is called ‘proper-orthogonal-decomposition’ (POD)^27–29^, but in other domains it is known as principal-component analysis or the Karhunen–Loève expansion.

In case of (non-linear) elastic simulations, only a few POD modes are necessary to accurately represent the displacement fields, but many more modes must be incorporated in case of elastoplastic simulations to achieve a similar accuracy as for elastic simulations - albeit this depends amongst others on the amount of elastoplastic deformation that occurs. To reduce the number of modes for elastoplastic analyses, the modes may be adaptively changed during the course of a reduced-order-model simulation^30,31^, but this is not considered in the current contribution (nor in the previous RNN-POD frameworks^24–26^).

Returning to the few RNN-POD surrogates^24–26^ in which the modes do not evolve during the course of a simulation/emulation (but only the coefficients), it may be concluded that their capabilities and limitations are not yet fully investigated (nor that a consensus about their potential is reached).

For instance, the studies of Cao et al.^24^ and Freitag et al.^25^ strongly focus on the application of mechanical tunnelling, making it hard to distinguish (1) if large (plastic) deformations and rotations are considered, (2) if substantial plasticity-induced localizations occur, if these localizations occur at different locations throughout the domain for different scenarios, and (3) the range of variations for which the framework was trained. Predecessors to these studies^24,25^, e.g. Nagel et al.^32^, also did not reveal these points.

On the other hand, the recent study of Im et al.^26^ clearly mentions the limitation to small deformations and rotations. Furthermore, plasticity-induced localization only appears at one or a few location(s) that do not change during the course of the simulations. Moreover, the applied boundary conditions seem to be limited to a single (history-dependent) ‘variable’ (e.g. a horizontal excitation or a vertical force). The numerical limitations of the investigation^26^ are furthermore characterized by the fact that only eight modes suffice to obtain highly accurate results.

### Aim

The aim of the current contribution is to numerically reveal the capabilities and limitations of RNN-POD frameworks, by considering:

1. the entire, current displacement field as the output of interest,
2. a relatively simple RNN (with a single central layer of gated recurrent units (GRUs), sandwiched between two layers of conventional neurons),
3. large deformations with plasticity-induced localizations at several locations in the domain, as well as large variations of the imposed deformations, and
4. the application to RVE computations with periodic boundary conditions in order to serve multiscale analyses.

However, embedding the RNN-POD framework in a multiscale simulation is not considered yet. Also, the accuracy of the consistent tangent moduli is not quantatively considered yet—only the accuracy of the macroscale stress-deformation response, and the accuracy of the microstructural field of the plastic multiplier.

### Outline

The remainder of this paper is organized as follows. The next section concisely discusses how to efficiently train the surrugate for an RVE, by removing the rigid body rotations from the macroscale deformation gradient tensor (which is imposed on the boundaries of the RVE). The direct numerical simulations are concisely discussed in section "Direct numerical simulations". Section "POD-based model-order-reduction" describes a conventional POD-based reduced-order-model (ROM) followed by the description of the neural network architecture in section "Recurrent neural network". Section "Data collection" discusses the generation of training data. The results are divided over two sections: the results of the RNN are presented in section "RNN results" and the mechanical results, which are of course of primary interest, are presented in section "Mechanical results". A conclusion is presented in section "Conclusion".

## Removing rigid body rotations from the RVE and its surrogate

Surrogate models based on neural networks require a substantial amount of training data. The rather slow, conventional POD-based ROM (which in turn also needs training data, but substantially less than the RNN-POD surrogate), must generate this large amount of data. Therefore, it is convenient if the amount of training data can be restricted as much as possible.

In the case of RVE computations, which are governed by an applied macroscopic deformation, it is more efficient to train the emulator for ‘pure’ macroscopic deformations (meaning that rigid body rotations are extracted from the macroscopic deformation) than for the actual macroscopic deformations. Extracting the rigid body rotations from the macroscale deformation reduces the number of inputs of the RNN-POD emulator from four to three in 2D (and from nine to six in 3D). The current section shows that the ‘pure’ macroscale stress-deformation relation (i.e. *without* rigid body rotations) can be rapidly transformed into the actual macroscale stress-deformation relation (*with* rigid body rotations).

### Macroscopic stress-deformation relation

First-order multiscale (FE$^\text {2}$) simulations comprise interwoven simulations at two length scales^33–36^. The macroscale simulation, with its modelling domain at the size of the entire application or product, uses an implicit constitutive relation (as well as its sensitivity), which is obtained from microstructural RVE simulations (with its modelling domain at the size of the microstructure). The macroscale deformation gradient tensor at time *t*, ${\textbf{F}}^M(t)$ (at a quadrature point of the macroscale discretization), is imposed on an RVE. This results in a displacement field within the RVE, in turn yielding a stress field inside the RVE. The macroscale (homogenized) 1$\text {st}$ Piola Kirchoff (PK) stress tensor, ${\textbf{P}}^M({\textbf{F}}^M(t),{\textbf{z}}^\mu(t))$ (which depends on the macroscale deformation gradient tensor and on a yet unspecified number of plastic variables), can be computed using the field of the microstructural 1$\text {st}$ (PK) stress tensors that occurs inside the RVE, ${\textbf{P}}^\mu ({\textbf{F}}^\mu ({\overrightarrow{X}}^\mu ,t),{\textbf{z}}^\mu ({\overrightarrow{X}}^\mu ,t))$, as follows:

1 $$\begin{aligned} {\textbf{P}}^M({\textbf{F}}^M,{\textbf{z}}^M)=\frac{1}{V_0}\int _{V_0}{\textbf{P}}^\mu ({\textbf{F}}^\mu ({\overrightarrow{X}}^\mu ),{\textbf{z}}^\mu ({\overrightarrow{X}}^\mu ))\text {d}V_0, \end{aligned}$$

where ${\textbf{F}}^\mu$ and ${\overrightarrow{X}}^\mu$ denote the (microstructural) deformation gradient tensor in the RVE and the reference location vector in the RVE, respectively. $V_0$ denotes the reference volume of the RVE (i.e. area in 2D).

As mentioned before, the implicit relation ${\textbf{P}}^M({\textbf{F}}^M(t),{\textbf{z}}^M(t))$ drives the simulation at the macroscale. The sensitivity of the macroscale stress with respect to the macroscale deformation gradient tensor, i.e. $\frac{\partial {\textbf{P}}^M}{\partial {\textbf{F}}^M}$, is therefore also required at the macroscale.

The issue of interest here is that macroscale deformation gradient tensor ${\textbf{F}}^M$ embeds rigid body rotations and we want to extract them from the deformation (and consequently, also from the macroscale stress). To this purpose, we multiplicatively decompose the macroscale deformation gradient tensor at time *t* in a (proper orthogonal) rotation tensor, ${\textbf{R}}^M(t)$ and a (symmetric) ‘pure’ deformation gradient tensor, ${\textbf{U}}^M(t)$, according to:

2 $$\begin{aligned} {\textbf{F}}^M={\textbf{R}}^M\cdot {\textbf{U}}^M, \end{aligned}$$

where ($\cdot$) denotes an inner product.

Consequently, if a pure macroscopic deformation, ${\textbf{U}}^M(t)$, is imposed to the RVE (instead of ${\textbf{F}}^M(t)$), the resulting macroscale stress tensor, $\tilde{{\textbf{P}}}^M({\textbf{U}}^M(t),{\textbf{z}}^M(t))$ (which is still obtained according to the volume average in Eq. (1)), is related to the macroscale stress tensor that embeds rigid body rotations, i.e. ${\textbf{P}}^M({\textbf{F}}^M(t),{\textbf{z}}^M(t))$, as follows:

3 $$\begin{aligned} {\textbf{P}}^M={\textbf{R}}^M\cdot \tilde{{\textbf{P}}}^M. \end{aligned}$$

The sensitivities of the two stress tensors with respect to the macroscale deformation gradient tensor are related as follows:

4 $$\begin{aligned} \frac{\partial {\textbf{P}}^M}{\partial {\textbf{F}}^M}=\left( \tilde{{\textbf{P}}}^M\cdot \left( \frac{\partial {\textbf{R}}^M}{\partial {\textbf{F}}^M}\right) ^{T_{12}}\right) ^{T_{12}}+{\textbf{R}}^M\cdot \frac{\partial \tilde{{\textbf{P}}}^M}{\partial {\textbf{U}}^M}:\frac{\partial ({\textbf{U}}^M)^T}{\partial {\textbf{F}}^M}, \end{aligned}$$

where superscript *T* denotes the transpose, superscript $T_{12}$ denotes a conjugate transpose where the first and second base vectors change sequence, and ( : ) denotes the double inner product.

This indicates that if the RVE is exposed to macroscale deformations without rigid body rotations, the resulting macroscale stress tensor (as well as its sensitivity) can straightforwardly be related to the relevant macroscale stress tensor (as well as its sensitivity) that is affected by rigid body rotations.

## Direct numerical simulations

In the current section, we discuss the RVE simulations. The RVE simulations are the direct numerical simulations (DNS) which the POD-based ROM, and particularly the RNN-POD emulator must mimic.

**Figure 1:**
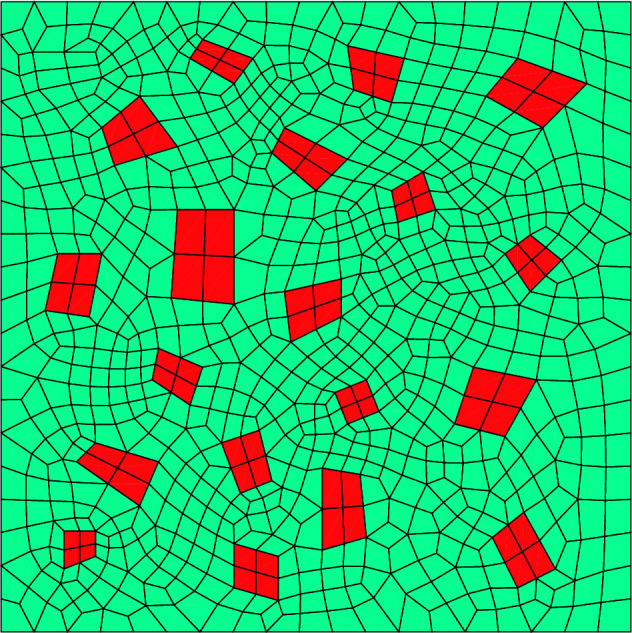
The meshed RVE with the elastoplastic matrix in green and the stiff, elastic particles in red.

The DNS are 2D RVE computations with stiff, elastic particles embedded in an elastoplastic matrix (see Fig. 1). In relation to the previous section, this entails that our simulations are microstructural simulations (superscript $\mu$ in the previous section) and the macroscale (superscript *M* in the previous section) is completely neglected. Thus, unless specified otherwise, all quantities discussed in the current section are microstructural quantities, but superscript $\mu$ is omitted in order to ease the notation.

### Constitutive model

We consider plane strain conditions and employ isoparametric bilinear quadrilateral finite elements with four quadrature points. An *F*-bar method is utilized to alleviate locking due to the incompressibility of the plastic deformation. Within the *F*-bar framework, the volume change of the deformation at a quadrature point is replaced with the volume change at the center of the element. Thus, the deformation gradient tensor that is considered for the constitutive model in each quadrature point, $\bar{{\textbf{F}}}$, is written as:

5 $$\begin{aligned} \bar{{\textbf{F}}}=\frac{J_c^{\frac{1}{3}}}{J^{\frac{1}{3}}}{\textbf{F}}, \end{aligned}$$

where ${\textbf{F}}$ denotes the deformation gradient tensor in the quadrature point, $J=\text {det}({\textbf{F}})$ the volume change in the quadrature point, and $J_c$ the volume change in the center of the element.

The deformation gradient tensor that is used in the actual constitutive model, $\bar{{\textbf{F}}}$, is multiplicatively decomposed into an elastic (subscript *e*) and a plastic (subscript *p*) deformation gradient tensor:

6 $$\begin{aligned} \bar{{\textbf{F}}}={\textbf{F}}_e\cdot {\textbf{F}}_p. \end{aligned}$$

The $1^{\text {st}}$ Piola-Kirchhoff (PK) stress tensor is defined according to the following derivative:

7 $$\begin{aligned} {\textbf{P}}=\frac{\partial W}{\partial {\textbf{F}}_e}, \end{aligned}$$

with the following strain energy density:

8 $$\begin{aligned} W=\frac{E(I_e-3-2\text {ln}(J_e))}{4(1+\nu )}+\frac{E\nu (\text {ln}(J_e))^2}{2(1+\nu )(1-2\nu )}, \end{aligned}$$

where *E* and $\nu$ denote Young’s modulus and Poisson’s ratio, respectively. Furthermore: $I_e=\text {tr}({\textbf{F}}_e^T\cdot {\textbf{F}}_e)$ and $J_e=\text {det}({\textbf{F}}_e)$.

In order to assess if plastic yielding occurs, we first relate the $1^{\text {st}}$ PK stress tensor to the Mandel stress, ${\textbf{M}}$, according to:

9 $$\begin{aligned} {\textbf{M}}={\textbf{F}}_e^T\cdot {\textbf{P}}, \end{aligned}$$

then we compute the deviatoric Mandel stress according to:

10 $$\begin{aligned} {\textbf{M}}^{dev}={\textbf{M}}-\frac{\text {tr}({\textbf{M}})}{3}{\textbf{I}}, \end{aligned}$$

(where ${\textbf{I}}$ denotes the identity tensor) and subsequently, we use the deviatoric Mandel stress in the following von Mises-type yield function:

11 $$\begin{aligned} y=\sqrt{\frac{3}{2}{\textbf{M}}^{dev}:{\textbf{M}}^{dev}}-M_0-h\,\lambda ^m, \end{aligned}$$

where material parameters $M_0$, *h* and *m* denote the initial yield stress, the hardening modulus and an exponential hardening parameter, respectively. Furthermore, $\lambda$ denotes the plastic multiplier, with an initial value of zero.

The evolution of the plastic multiplier is linked to the evolution of the plastic deformation according to the following associated flow rule:

12 $$\begin{aligned} \dot{{\textbf{F}}}_p={\dot{\lambda }}\frac{\partial \,y}{\partial {\textbf{M}}}\cdot {\textbf{F}}_p. \end{aligned}$$

The Karush-Kuhn-Tucker conditions close the constitutive model:

13 $$\begin{aligned} y\le 0, \quad \quad \quad \quad {\dot{\lambda }}\ge 0, \quad \quad \quad \quad {\dot{\lambda }}y=0. \end{aligned}$$

The same constitutive description is used for the elastic particles and the elastoplastic matrix. However, in order to guarantee that the particles only deform elastically, their initial yield stress ($M_0$) is set to a substantially large value that will never be reached during the simulations. Furthermore, Young’s modulus of the elastic particles is set to twenty times that of the elastoplastic matrix. The actual parameter values are presented in section 6.

### Periodic RVE simulations

Because we are interested in RVE simulations, the boundary conditions are dictated by the ‘pure’ macroscale deformation, ${\textbf{U}}^M(t)$. The fact that the pure macroscale deformation depends on pseudo-time *t*, not a single ${\textbf{U}}^M$ is provided, but the entire sequence for all time increments.

The current boundary conditions of the RVE simulations are governed by the current, ‘pure’ deformation gradient tensor, ${\textbf{U}}^M$. We impose the macroscale pure deformation via periodic boundary conditions. This entails that the displacement of a node on the bottom boundary (node *a*) is linked to the displacement of a node on the top boundary (node *b*) according to:

14 $$\begin{aligned} {\overrightarrow{u}}^b-{\overrightarrow{u}}^a=\left( {\textbf{U}}^M-{\textbf{I}}\right) \cdot \left( {\overrightarrow{X}}^b-{\overrightarrow{X}}^a\right) , \end{aligned}$$

where ${u}$ and ${\overrightarrow{X}}$ denote a nodal displacement and a nodal reference location, respectively. The same constraints are present between nodes on the left boundary and nodes on the right boundary. To enable the application of the displacement constraints between pairs of nodes on opposing boundaries, we employ a periodic mesh (see Fig. 1).

In the RVE simulations, the left bottom node obtains displacement boundary conditions of zero. This entails that the displacements of the other corner nodes are known and can thus also be prescribed using displacement boundary conditions. However, the displacements of the remaining nodes on the boundaries, are not known in advance and hence, the aforementioned constraint must be included as an actual constraint (in fact, as two scalar constraints in a 2D setting as here). We have chosen to enforce the constraints using Lagrange multipliers.

After linearization, the DNS implies that the following system of linear equations must be constructed and solved for each iteration, for each time increment:

15 $$\begin{aligned}{} & {} \begin{bmatrix}\underline{\underline{K}}_{int}(\underline{u},\underline{z})&{} \left( \frac{\partial \underline{c}}{\partial \underline{u}}\right) ^T\\ \frac{\partial \underline{c}}{\partial \underline{u}} &{} \underline{\underline{0}}\end{bmatrix}\begin{bmatrix}d\underline{u}\\ d\underline{g}\end{bmatrix} \nonumber \\{} & {} \quad =\begin{bmatrix}\underline{f}_{ext}-\underline{f}_{int}(\underline{u},\underline{z})-\underline{g}^T\frac{\partial \underline{c}}{\partial \underline{u}}\underline{c}(\underline{u})\\ \underline{c}(\underline{u})\end{bmatrix}, \end{aligned}$$

where column $\underline{u}$ collects the displacement components of all nodes at an intermediate solution, column $\underline{z}$ the plastic variables in all quadrature points ($\lambda$ and the components of ${\textbf{F}}_p$), column $\underline{g}$ the Lagrange multipliers, column $\underline{c}$ the constraints due to the periodic boundary conditions, column $\underline{f}_{ext}$ the components of the reaction forces, column $\underline{f}_{int}$ the components of the internal forces and matrix $\underline{\underline{K}}_{int}$ the derivatives of the internal force components with respect to the displacement components. $d\underline{u}$ and $d\underline{g}$ denote the corrections to $\underline{u}$ and $\underline{g}$, which are to be computed.

## POD-based model-order-reduction

Now that the DNS are discussed, we continue the current section with the reduced-order model (ROM). The ROM is not the end goal; it only serves to reduce the number of output variables that will be emulated by the final framework, i.e. the RNN-POD surrogate.

Projection-based model-order-reduction (MOR) projects the original $n_u$ kinematic variables, $\underline{u}$, onto the $n_m$ variables of the ROM (${\underline{\alpha }}$) using $n_m$ modes according to:

16 $$\begin{aligned} \underline{u} \approx \sum _{i=1}^{n_m}{\underline{\phi }}_i\alpha _i=\underline{{\underline{\Phi }}}\,{\underline{\alpha }}, \end{aligned}$$

where ${\underline{\phi }}_i$ of length $n_u$ denotes the $i^{\text {th}}$ mode and scalar $\alpha _i$ denotes its associated modal coefficient that is to be computed by the ROM (whereas the final RNN-POD surrogate will rapidly emulate these modal coefficients). $\underline{{\underline{\Phi }}}$ and ${\underline{\alpha }}$ collect all the modes and the modal coefficients in an $n_u \times n_m$ snapshot matrix and a column of length $n_m$, respectively.

In the method of proper-orthogonal-decomposition (POD), modes $\underline{{\underline{\Phi }}}$ are the left-singular (orthonormal) vectors corresponding to the largest singular values of an $n_u \times n_s$ matrix that stores all relevant $n_s$ training solutions (as columns) computed by the DNS. In other words, the ROM needs training data predicted by the DNS, whereas the final RNN-POD surrogate needs training data predicted by the ROM. However, the ROM requires much less training data than the RNN-POD surrogate.

In RVE simulations, the modes are not directly extracted from the training solutions (computed by the DNS). Rather, each solution field is first decomposed into a homogeneous (i.e. affine) field, $\bar{\underline{u}}$, and a fluctuating field, $\tilde{\underline{u}}$:

17 $$\begin{aligned} \underline{u}=\bar{\underline{u}}+\tilde{\underline{u}}. \end{aligned}$$

Because the macroscopic deformation is applied to the boundaries of the RVE, homogeneous field $\bar{\underline{u}}$ is known. The modes are thus only used to interpolate the fluctuating part of the displacement field, yielding the following expression:

18 $$\begin{aligned} \underline{u}=\underline{{\underline{\Psi }}}\,{\underline{\omega }}+\underline{{\underline{\Phi }}}\,{\underline{\alpha }}, \end{aligned}$$

where ${\underline{\omega }}$ of length 3 (in 2D) is completely dictated by the (known) components of ${\textbf{U}}^M$ (three components in 2D simulations) and $\underline{{\underline{\Psi }}}$ (of size $n_u \times 3$ in 2D) homogeneously interpolates the displacement components.

According to Eqs. (17) and (18), the homogeneous deformations are first subtracted from the training solutions, and SVD is applied to the resulting (i.e. fluctuating) displacement field.

The decomposition generates modes that are themselves periodic and hence, periodicity does not need to be actively enforced in the ROM (nor in the RNN-POD surrogate). Consequently, the system of linear equations that needs to be solved in terms of update $d{\underline{\alpha }}$ in the online POD reduced-order-model reads:

19 $$\begin{aligned} \begin{aligned} \underline{{\underline{\Phi }}}^T\underline{\underline{K}}_{int}\,\underline{{\underline{\Phi }}}\,d{\underline{\alpha }} = -\underline{{\underline{\Phi }}}^T\underline{f}_{int} -\underline{{\underline{\Phi }}}^T\underline{\underline{K}}_{int}\,\underline{{\underline{\Psi }}}\,d{\underline{\omega }}, \end{aligned} \end{aligned}$$

where $d{\underline{\omega }}=\underline{0}$ during Newton’s method.

A relevant note must be made with regards to the use of projection-based MOR for elastoplastic simulations. The reason is that POD-based MOR requires only a few modes (five to twenty) for (non-linear) elastic simulations to obtain a good accuracy, but a substantially larger number of modes for elastoplastic simulations. In fact, studies as those of Zhang et al.^30^ and Daniel et al.^31^ adaptively change the modes during the course of an elastoplastic ROM simulation in order to reduce the number of modes (and hence, the number of degrees of freedom; the $n_m$ coefficients in ${\underline{\alpha }}$).

It is furthermore worth noting that the discussed ROM strategy only reduces the number of kinematic variables relative to the DNS (from $n_u$ variables in the DNS to $n_m$ variables in the ROM). This entails that *solving* the systems of linear equations is faster (cf. Eq. (15) and Eq. (19)). However, the computational efforts for *constructing* the systems of linear equations remains the same. In order to accelerate the construction of the systems, hyperreduction-type strategies may be utilized^37–39^, but our ROM does not exploit them.

## Recurrent neural network

In this section, we discuss the recurrent neural network (RNN) that rapidly emulates the model coefficients, ${\underline{\alpha }}$, for each increment. This circumvents the iterative process required for the conventional ROM of the previous section (see Eq. (19)). However, the conventional ROM must be performed many times beforehand, in order to generate the training data for the RNN.

### Network architecture

**Figure 2:**
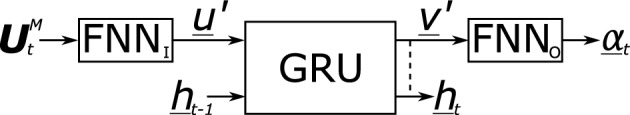
Schematic of the RNN. The three components of ${\textbf{U}}^M_t$ (at time increment *t*) are the input variables and the $n_m$ coefficients of ${\underline{\alpha }}_t$ (at time increment *t*) are the output variables. FNN$_I$ and FNN$_O$ are each a single layer feed-forward neural network. The GRU’s input is the output of $FNN_I$ (denoted by $\underline{u}'$), as well as the hidden states the GRU has predicted for the previous time increment, $\underline{h}_{t-1}$ (i.e. at time $t-1$). The GRU’s primary output, $\underline{v}'$, serves as the input for FFN$_O$, as well as updated hidden states, $\underline{h}_t$, that the GRU will use as input for the next time increment, i.e. at $t+1$.

The overall architecture of the employed RNN is graphically presented in Fig. 2 and is the same as that of Wu et al.^9^. It consists of a gated recurrent unit (GRU), sandwiched between two feed-forward neural networks (FNNs). The input, i.e. the three independent components of ${\textbf{U}}^M_t$ at time *t*, is fed to FNN$_I$, which transforms it into $\underline{u}'$. The output of FNN$_I$, $\underline{u}'$, together with the hidden states of the previous increment, $\underline{h}_{t-1}$, is transformed by the GRU into output $\underline{v}'$ and the next hidden states, $\underline{h}_{t}$. In turn, FNN$_O$ takes the GRU’s output, $\underline{v}'$ and transforms it into the modal coefficients of the current time increment, i.e. ${\underline{\alpha }}_t$.

Thus, the RNN outputs are evaluated from the problem inputs (${\textbf{U}}^M_t$) and from the hidden states of the previous increment ($\underline{h}_{t-1}$). These hidden states are updated by the RNN for the evaluation of the next increment ($\underline{h}_t$). The user can choose the number of hidden states that the RNN emulates during its training. These auxiliary quantities, i.e. the hidden states, play the role of internal state variables in constitutive models although they lack a physical meaning. Instead, they correspond to a reduced version of the internal variables of the physical problem.

Relevant to realize is that the two sets of output of the GRU, i.e. the output of interest to the current increment ($\underline{v}'$) and the updated hidden states necessary for the next increment ($\underline{h}_t$), are one and the same, i.e. $\underline{v}'=\underline{h}_t$. This entails that the GRU must emulate the same quantity in such a way that (i) quantity $\underline{v}'$ is accurate for the current increment, and (ii) the same quantity ($\underline{v}'=\underline{h}_t$) is accurate for the next increment. It may be clear that this limits the capabilities of the GRU to some extent. For instance, the RNN of Liu et al.^15^ and Bhattacharya et al.^16^ uses two independent quantities: one for the output of interest to the current increment (the equivalent to $\underline{v}'$) and one for the hidden states of interest to the next increment (the equivalent to $\underline{h}_t$, $\underline{v}'\ne \underline{h}_t$). Nevertheless, the GRU as exploited in the current contribution was demonstrated to be sufficiently accurate to emulate macroscale stress-deformation relations by Wu et al.^9^.

**Figure 3:**
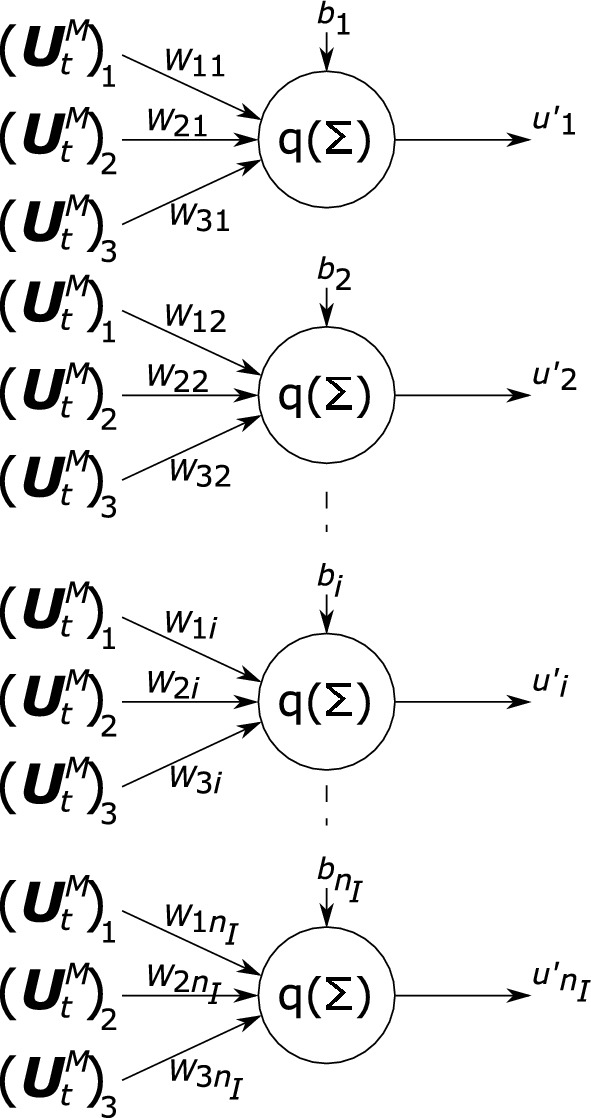
Schematic of FNN$_I$, that consists of a single layer. The three components of ${\textbf{U}}^M_t$ (at time increment *t*) are the input variables and the $n_I$ variables in $\underline{u}'$ are the output variables.

The architecture of both FNNs is rather straightforward, because they both consist of a single layer of neurons. This is depicted in Fig. 3 for FNN$_I$. All neurons use the leaky ReLU activation function. This entails that the $i^\text {th}$ output of FNN$_I$, $u_i'$, reads:

20 $$\begin{aligned} u_i'=\text {ReLU}( W_{1i}({\textbf{U}}^M_t)_1+W_{2i}({\textbf{U}}^M_t)_2+W_{3i}({\textbf{U}}^M_t)_3+b_i), \end{aligned}$$

where the three components of ${\textbf{U}}^M_t$ are denoted by $({\textbf{U}}^M_t)_1$, $({\textbf{U}}^M_t)_2$ and $({\textbf{U}}^M_t)_3$. $W_{1i}$, $W_{2i}$ and $W_{3i}$ denote weights that are to be identified (i.e. ‘trained’, ‘learned’). Bias $b_i$ must also be identified. The leaky ReLU activation function reads:

21 $$\begin{aligned} \text {ReLU}(x)=\text {max}(0.1x,x). \end{aligned}$$

The relation between the entire output of FNN$_I$ and its entire input may then be written as:

22 $$\begin{aligned} \underline{u}'=\text {ReLU}( \underline{\underline{W}}^T\underline{U}^M_t+\underline{b}), \end{aligned}$$

with:

23 $$\begin{aligned} \underline{U}^M_t=\begin{bmatrix} ({\textbf{U}}^M_t)_1 \\ ({\textbf{U}}^M_t)_2 \\ ({\textbf{U}}^M_t)_2\end{bmatrix}, \quad \underline{b}=\begin{bmatrix}b_1 \\ b_2 \\ ... \\ b_{n_I} \end{bmatrix}, \quad \underline{\underline{W}}=\begin{bmatrix}W_{11} &{} W_{12} &{}... &{} W_{1n_I}\\ W_{21} &{} W_{22} &{}... &{} W_{2n_I} \\ W_{31} &{} W_{32} &{}... &{} W_{3n_I} \end{bmatrix}. \end{aligned}$$

The leaky ReLU activation function in Eq. (22) must be applied to each component of the column independently.

**Figure 4:**
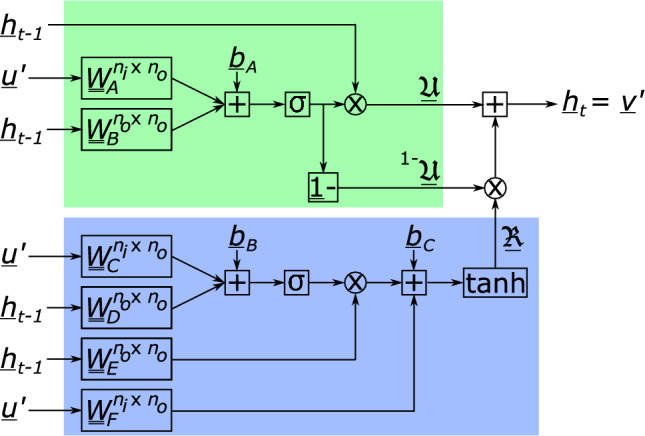
Schematic of the gated recurrent unit (GRU). Input $\underline{u}'$ is the output of FNN$_I$ and the GRU’s output, $\underline{v}'$, is the input of FNN$_O$. Output $\underline{v}'$ equals the hidden states for the next calculation of the RNN, $\underline{h}_t$. The reset gate is presented in blue (with output $\underline{\mathfrak {R}}$) and the update gate in green (with outputs $\underline{\mathfrak {U}}$ and ${}^{1-}\underline{\mathfrak {U}}$).

FNN$_I$’s output, $\underline{u}'$, is the input for the GRU. The GRU, graphically presented in Fig. 4, transforms the input together with the previous hidden states, $\underline{h}_{t-1}$, into output $\underline{v}'$, as well as into the next hidden states, $\underline{h}_t$. We stress again that the GRU’s output and its updated hidden states are the one and same ($\underline{v}'=\underline{h}_t$).

Fig. 4 illustrates that the GRU has a so-called ‘reset gate’ (blue) and an ‘update gate’ (green). The relations between the two outputs of the update gate and the input and the previous hidden states read:

24 $$\begin{aligned}{} & {} \underline{\mathfrak {U}}=\underline{h}_{t-1}\otimes \sigma \left( \underline{\underline{W}}^T_A\underline{u}'+\underline{\underline{W}}^T_B\underline{h}_{t-1}+\underline{b}_A\right) , \end{aligned}$$

25 $$\begin{aligned}{} & {} \quad {}^{1-}\underline{\mathfrak {U}}=\underline{1}-\sigma \left( \underline{\underline{W}}^T_A\underline{u}'+\underline{\underline{W}}^T_B\underline{h}_{t-1}+\underline{b}_A\right) , \end{aligned}$$

where matrices $\underline{\underline{W}}$ and column $\underline{b}_A$ denote matrices with weights and a column with biases, respectively. All their components must be trained/identified/learned. Furthermore, $\sigma (\bullet )$ denotes the sigmoid activation function:

26 $$\begin{aligned} \sigma (x)=\frac{1}{1+\text {exp}(-x)}, \end{aligned}$$

which must be applied to each component of the column independently. A multiplication indicated by $\otimes$ between columns $\underline{a}$ and $\underline{b}$ furthermore means that the $i^\text {th}$ component of resulting column $\underline{c}$ ($\underline{c}=\underline{a}\otimes \underline{b}$), is calculated as $c_i=a_ib_i$.

On the other hand, the relation between the input, the previous hidden states and the output of the reset gate reads:

27 $$\begin{aligned} \underline{\mathfrak {R}}=\text {tanh}\bigg (\sigma \left( \underline{\underline{W}}_C^T\underline{u}'+\underline{\underline{W}}_D^T\underline{h}_{t-1}+\underline{b}_B\right) \otimes (\underline{\underline{W}}_E^T\underline{h}_{t-1})+\underline{\underline{W}}_F^T\underline{u}'+\underline{b}_C\bigg ), \end{aligned}$$

where $\text {tanh}(\bullet )$ denotes the hyperbolic tangent activation function:

28 $$\begin{aligned} \text {tanh}(x)=\frac{\text {exp}(x)-\text {exp}(-x)}{\text {exp}(x)+\text {exp}(-x)}, \end{aligned}$$

which means for Eq. (27) that it must be applied to each component of the column independently.

Finally, the GRU’s output of current interest, which equals the updated hidden states, reads:

29 $$\begin{aligned} \underline{v}'=\underline{h}_t=\underline{\mathfrak {U}}+\underline{\mathfrak {R}}\otimes {}^{1-}\underline{\mathfrak {U}}. \end{aligned}$$

The new hidden states, $\underline{h}_t$, are stored for the next time that the RNN is called upon (since they play a similar role as internal variables in elastoplastic simulations). On the other hand, the GRU’s output serves as the input of FNN$_O$, which only contains a single layer of neurons (employing the leaky ReLU activation function). Consequently, FNN$_O$ must contain the same number of neurons as the number of modal coefficients in ${\underline{\alpha }}$, i.e. $n_m$. (Note that $n_O$, denoting the number of hidden states as well as the number of output variables of the GRU, is independent of $n_m$.)

### Learning phase

Now the RNN is formulated, its parameters (i.e. the weights and biases) must be identified (i.e. ‘learned’ or ‘trained’ in NN terminology). The number of parameters is substantial, because FNN$_I$ and FNN$_O$ each comes with a weight matrix and a column of biases (i.e. $4n_I$ for FNN$_I$ and $(n_O+1)n_m$ for FNN$_O$). Furthermore, the GRU comes with six weight matrices and three columns with biases (i.e. $(3n_O+3n_I+3)n_O$). The total number of parameters is thus $4n_I+n_m+(n_m+3n_O+3n_I+3)n_O$ (where once again, $n_I$ denotes the number of neurons in FNN$_I$, $n_O$ the number of hidden states, which equals the number of neurons in FNN$_O$, and $n_m$ the number of coefficients, i.e. the length of ${\underline{\alpha }}$).

For the ease of the notation, we store all parameters of the RNN in column $\underline{p}$. First, the conventional reduced-order-model is performed numerous times and all coefficients predicted for each time increment, for each training simulation, are stored. Both input variables (${\textbf{U}}^M$) and output variables (${\underline{\alpha }}$) are then normalised in view of training the RNN. We will denote the normalized output variables as $\underline{\breve{\alpha }}^i_t$, where superscript *i* indicates the training simulation number and subscript *t* refers to the time increment number. The RNN must replicate these coefficients. To this purpose, one could minimize the following least square error:

30 $$\begin{aligned} L(\underline{p}) = \frac{1}{n_sn_{incr}}\sum _{i=1}^{n_s}\sum _{t=1}^{n_{incr}}\Arrowvert \underline{\breve{\alpha }}^i_t-{\underline{\alpha }}^i_t(\underline{p},\underline{h}_{t-1}^i(\underline{p})) \Arrowvert ^2, \end{aligned}$$

where $n_s$ denotes the number of training simulations performed with the conventional ROM, $n_{incr}$ denotes the number of increments of each training simulation, and ${\underline{\alpha }}^i_t$ denotes the RNN’s prediction of the coefficients. Furthermore, $\Arrowvert \bullet \Arrowvert$ denotes the $L^2$-norm.

We use the ADAM optimizer^40^ to identify (i.e. ‘train’ or ‘learn’) the weights and biases (stored in $\underline{p}$). ADAM combines stochastic gradient descent with root mean square propagation. The stochastic gradient descent part of the optimizer requires the gradient of *L* with respect to parameters $\underline{p}$. Because the RNN’s prediction depends directly on parameters $\underline{p}$, but also indirectly, via the dependency on the previous hidden states ($\underline{h}_{t-1}^i$), this dependency must be incorporated in the gradient.

The stochastic part of the gradient descent algorithm implies that it does not consider the entire least squares error every iteration of the optimization. Instead, only a part of the $n_s$ training simulations are considered for each iteration of the optimization, which is called a batch. Thus, if ten batches are considered, it takes ten iterations to consider all $n_s$ training simulations in the optimization (which is called an epoch in NN terminology). Because of the dependency on the hidden states, the data of each training simulation (i.e. a sequence of $n_{incr}$ columns $\underline{\breve{\alpha }}$) must be present in one batch in its entirety. In fact, the correct sequence of the incremental data must be incorporated in the training.

Thus, the (stochastic) gradient determines in which direction the RNN’s parameter values are updated. On the other hand, the step size (‘learning rate’ in NN terminology), which governs how much the RNN’s parameter values are updated in the direction of the (stochastic) gradient, are determined by the first moment and second moment estimates of the root mean square part of ADAM.

## Data collection

Now all methodologies are discussed, the current section explains the setup of the DNS, as well as the generation of the training data. The previous sections have already mentioned that two types of training data are required for the RNN-POD emulator: the training of the ROM requires solution fields computed by the DNS, and the training of the RNN requires modal coefficients computed by the ROM. However, the RNN requires substantially more training data than the ROM.

### DNS model setup

The RVE of interest (see Fig. 1) consists of stiff elastic particles in an elastoplastic matrix. The mechanical parameters of the elastoplastic matrix are set to $E=1$, $\nu =0.3$, $M_0=0.01$, $h=0.02$ and $m=1.05$. The mechanical parameters of the stiff elastic particles are set to $E=20$ and $\nu =0.3$, while $M_0=10^{10}$ ensures that the particles behave purely elastically.

Because the matrix deforms mostly plastically and plastic deformation is isochoric, and because the particles deform only minimally relative to the matrix (due to the ratio of Young’s moduli), we only apply isochoric macroscale deformations (i.e. $\text {det}({\textbf{U}}^M)=1$), governed by bounds $0.75<U^M_{11}<1.25$, $0.75<U^M_{22}<1.25$ and $-0.25<U^M_{12}<0.25$. This means that it is sufficient to only consider $U^M_{11}$ and $U^M_{12}$ as the RNN’s input (since $\text {det}({\textbf{U}}^M)=1$, $U^M_{11}$ and $U^M_{12}$ dictate the value of $U^M_{22}$).

### Training the ROM

**Figure 5:**
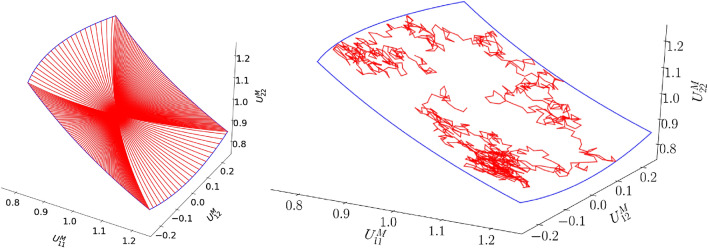
Left: Some cyclic load paths for training (red). Right: A random load path for training (red). The bounds of the load paths are presented by blue lines.

First, the modes of the conventional ROM are identified. To this purpose, 350 cyclic loading (left in Fig. 5) and 1,000 random loading simulations (right in Fig. 5) are performed using the DNS by fully prescribing the load paths. All simulations are performed using 1,000 increments but only 10 solutions per simulation are stored. After subtracting the homogeneous deformation of each solution, SVD is applied to the snapshot matrix (consisting of 13,500 solutions) and the left-singular vectors associated with the 100 largest singular values are used as modes (i.e. $n_m=100$).

The generation of the random load paths (for the random loading simulations) require some more explanation, because they are not entirely random (see the right diagram of Fig. 5 for the load path of a single random loading simulation). The reason is that the size of each increment (i.e. when the random load path changes direction) is exactly the same (a size of 0.02 in the $U_{11}^M-U_{12}^M-U_{22}^M$ space); only the direction of the load path is random.

Furthermore, random loading is not per se simulated because it is expected in nested multiscale simulations. Instead, it is considered to enhance the training because in true multiscale simulations with cyclic loading, the cyclic loading path of each RVE will be not be perfectly proportional and is different for each RVE in the macroscale discretization^9^.

### Training the RNN

After the ROM is trained, training data for the RNN must be generated, i.e. modal coefficients $\underline{\breve{\alpha }}_t$ in Eq. (30). In case of (hyper)elasticity, these modal coefficients can be directly obtained from the displacement fields predicted by the DNS. In our case of elastoplastic simulations however, even with 100 modes, the modal reconstruction of the displacement fields predicted by the DNS is not perfect. Consequently, the ROM must be used to create training data for the RNN.

The RNN’s training data is generated by performing 360 cyclic loading and 10,100 random loading simulations by the conventional POD-based MOR (with 100 modes and 1,000 increments). Of these ROM simulations, 350 cyclic loading simulations and 10,000 random loading simulations are used for the actual training (i.e. $n_s=10,350$). The remainder (i.e. 10 cyclic loading and 100 random loading ROM simulations) are used as verification simulations, meaning that they are used to assess whether the RNN is overfitting.

We would like to note that also the random load paths for the random ROM simulations are not entirely random. Similar as for the random loading simulations to train the ROM, the step size of each increment is the same (0.02 in the $U_{11}^M-U_{12}^M-U_{22}^M$ space).

Whereas only ten solutions per training simulation were used for the training of the conventional ROM (i.e. learning modes $\underline{{\underline{\Psi }}}$ in "Recurrent neural network section), the training of the RNN requires that the modal coefficients of all increments are stored. Furthermore, the sequence of the modal coefficients for all increments must be accounted for in the training.

Finally, we summarize the hyperparameters for the ADAM optimizer. Each mini-batch considers approximately 1% of the training data. In the ADAM optimizer, we set the initial learning rate to 0.001 (i.e. ‘$\gamma$’ in ADAM terminology) and the exponential decays for the first order and second order estimates to 0.9 and 0.999, respectively (‘$\beta ^1$’ and ‘$\beta ^2$’ in ADAM terminology, respectively). A cut-off value of $10^{-8}$ is used to prevent divisions by zero (‘$\epsilon$’ in ADAM terminology). The initial values for the weights and biases are randomly generated and all hidden states are initially set to -1 (as suggested by Wu et al.^9^).

## RNN results

We now investigate the accuracy of the RNN for (i) different numbers of layers in FFN$_O$, (ii) different numbers of neurons in FFN$_I$, and (iii) different numbers of hidden states (where the number of hidden states equals the number of neurons in each layer of FFN$_O$). We investigate 64 possible configurations. The number of layers in FFN$_O$ is varied between 1, 2, 3 and 4. The number of neurons in FFN$_I$ is varied between 100, 250, 500 and 1,000. The number of hidden states (and hence, the number of neurons in each layer of FFN$_O$) is varied between 400, 800, 1,600 and 2,500.

We investigate the accuracy of the 64 RNNs after they are trained for 60,000 epochs. After 60,000 epochs, the RNN with the smallest loss function is then assumed to be the best possible RNN from the available options. We train this final RNN for a total of 450,000 epochs after which we have observed that its loss function does not reduce further.

**Figure 6:**
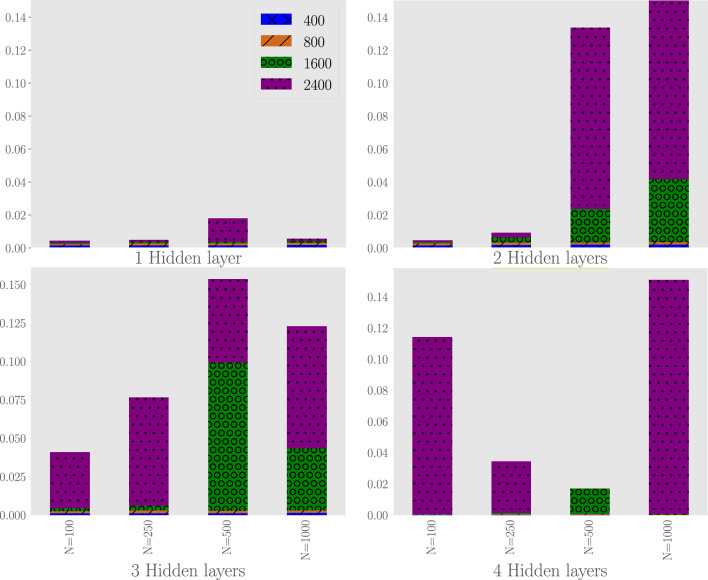
The loss function for the verification data after training for 60,000 epochs for three neural network parameters: (i) the number of hidden layers in the $FFN_O$ (top-left: 1 layer, top-right: 2 layers, bottom-left: 3 layers, bottom-right: 4 layers), (ii) the number of neurons in the single layer of $FFN_I$ (‘*N*’) and (iii) the number of hidden states (the different colors/patterns). The size of the bar corresponds to the value of the loss function (i.e. a large bar corresponds to a large value of the loss function). Please note that some loss functions are so small that they are difficult to observe.

The loss functions of the 64 possible RNNs after 60,000 epochs are presented as bar charts in Fig. 6. The RNN with the smallest loss function after 60,000 epochs is the one with 1 layer in FFN$_O$, 100 neurons in FFN$_I$ and 1,600 hidden states (and hence 1,600 neurons in FFN$_O$).

**Figure 7:**
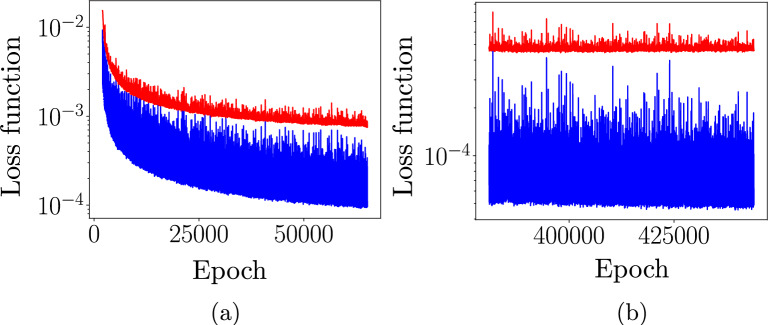
The evolution of the loss function for both training data (blue) and verification data (red) for the first 50,000 epochs (**a**) and the last 75,000 epochs for which it was trained (**b**). The loss function for the verification data (red) clearly does not reduce during the last 75,000 epochs (**b**).

The evolution of the loss function of this final RNN when trained for 450,000 epochs is presented on the left of Fig. 7 for the first 60,000 epochs and on the right of Fig. 7 for the last 75,000 epochs. It is clear that the loss function for the verification simulations does not decrease further.

**Figure 8:**
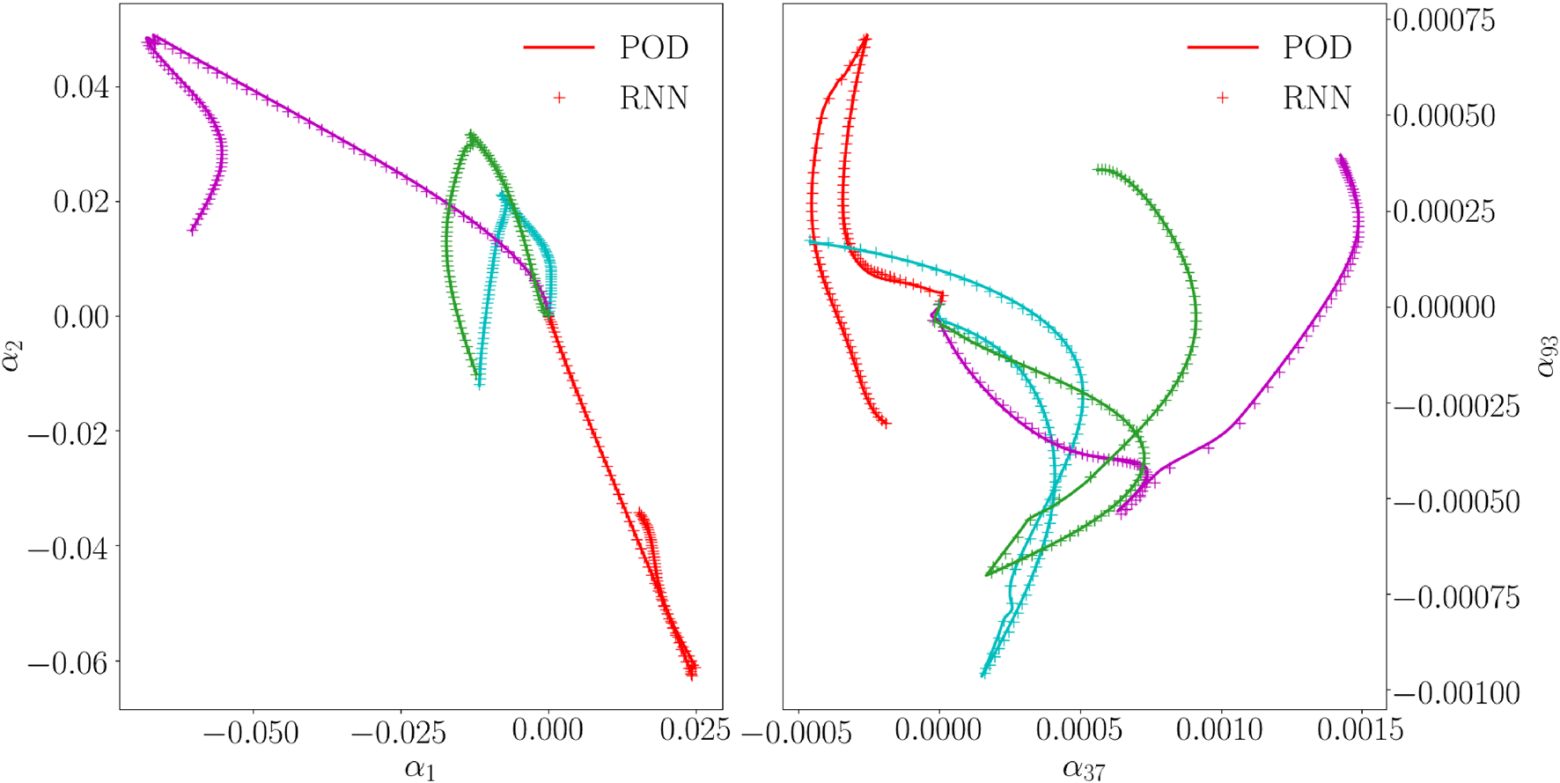
Cyclic loading verification simulations: Some RNN predictions (crosses) and the actual values (lines). The colors distinguish the four verification simulations.

**Figure 9:**
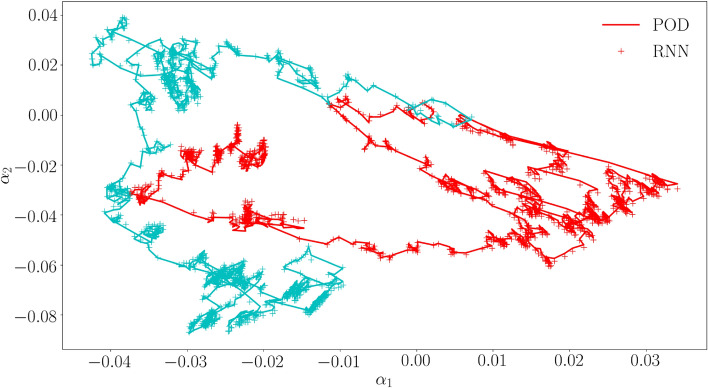
Random loading verification simulations: Some RNN predictions (crosses) and the actual values (lines). The colors distinguish two verification simulations.

The average error of the final RNN after training (defined in the same way as Eq. (30)) is $3\cdot 10^{-5}$ for the 10 cyclic verification loading simulations, and $4\cdot 10^{-4}$ for the 100 random loading simulations. In Fig. 8 a number of modal coefficients emulated by the final RNN are presented together with the training data for 4 cyclic verification loading simulations. The same is presented for 2 random loading simulations in Fig. 9.

## Mechanical results

Although the previous section has demonstrated the capabilities of the RNN, this does not automatically entail that the overall accuracy of the emulator is the same. In this section, the mechanical results of the RNN-POD surrogate are investigated and compared to those of the DNS and the conventional ROM. The comparison focuses on the macroscopic stress-deformation responses, but for the cyclic loading path, we also compare the microstructural field of the plastic multiplier at the end of the simulation/emulation ($\lambda$ in "POD-based model-order-reduction"section).

The comparison is divided in four parts: we first focus on the accuracy for a cyclic loading verification simulation/emulation, then on the accuracy for a random loading verification simulation/emultion, subsequently on the accuracy for three simulations/emulations for non-proportional load paths, and finally we compare the computational times.

### Cyclic loading

**Figure 10:**
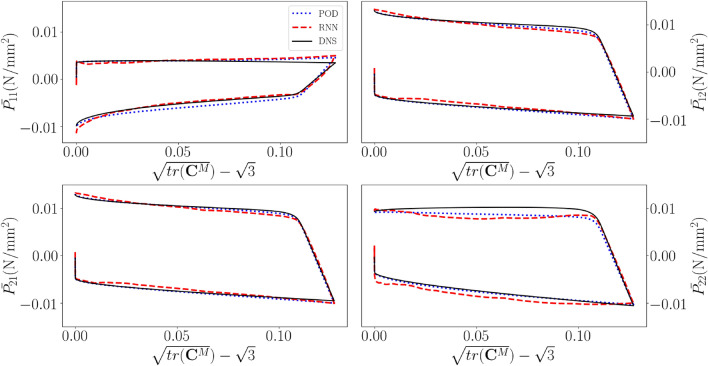
Macroscale, in-plane $1^{st}$ PK stress components as functions of the deformation for a cyclic loading verification simulation. Black: DNS results, dotted blue: conventional ROM results, dashed red: RNN-POD surrogate results.

Fig. 10 shows the macroscale $1\text {st}$ PK stress components for a cyclic verification simulation/emulation (that was not used to train the ROM, nor the RNN). We can see that the MOR results match those of the DNS fairly accurately (albeit not perfectly), indicating that the number of 100 modes is sufficiently large. The results of the RNN-POD surrogate also match those of the DNS and those of the MOR fairly accurately.

However, the problem is that the stress-deformation results emulated by the RNN-POD surrogate clearly fluctuate around the results of the ROM. This lack of smoothness, i.e. the fact that RNN-POD surrogate does not predict $\frac{\partial {\textbf{P}}^M}{\partial {\textbf{F}}_M}$ accurately, is expected to substantially affect the convergence behaviour of a true multiscale simulation.

**Figure 11:**
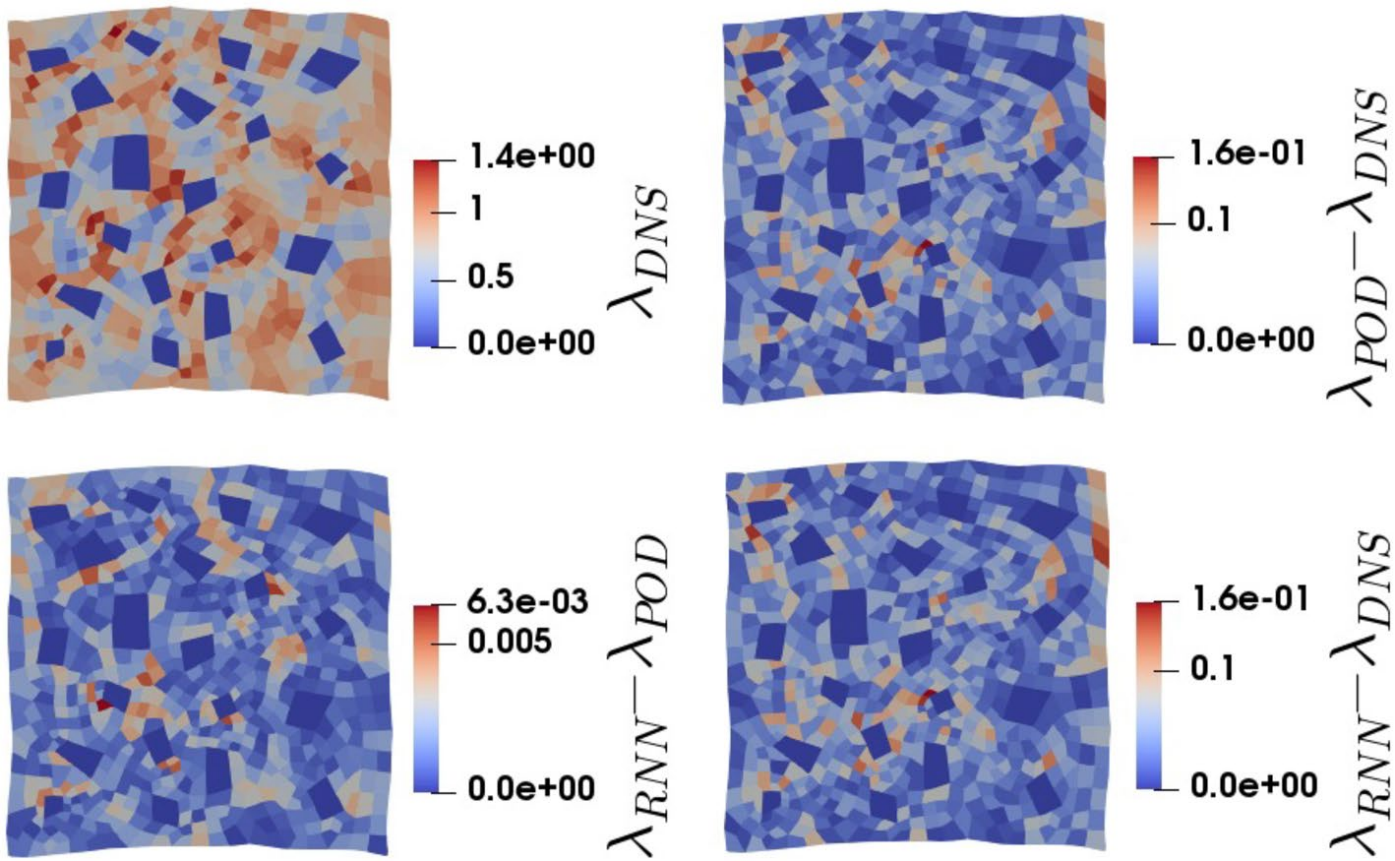
The plastic multiplier ($\lambda$) for a cyclic loading verification simulation. Top-left: the DNS results, top-right: the difference between the ROM results and the DNS results, bottom-left: the difference between the surrogate results and the ROM results, bottom-right; the difference between the surrogate results and the DNS results.

We nevertheless also study the accuracy of the RNN-POD surrogate for a microstructural field. To this purpose, the final fields of the plastic multiplier ($\lambda$) predicted by the different approaches for the same cyclic load case are presented in Fig. 11. The diagrams clearly show that the difference of the field emulated by the surrogate stems from the inaccuracy of the modal representation of the displacement field, and not from the inaccuracy of the RNN.

### Random loading

**Figure 12:**
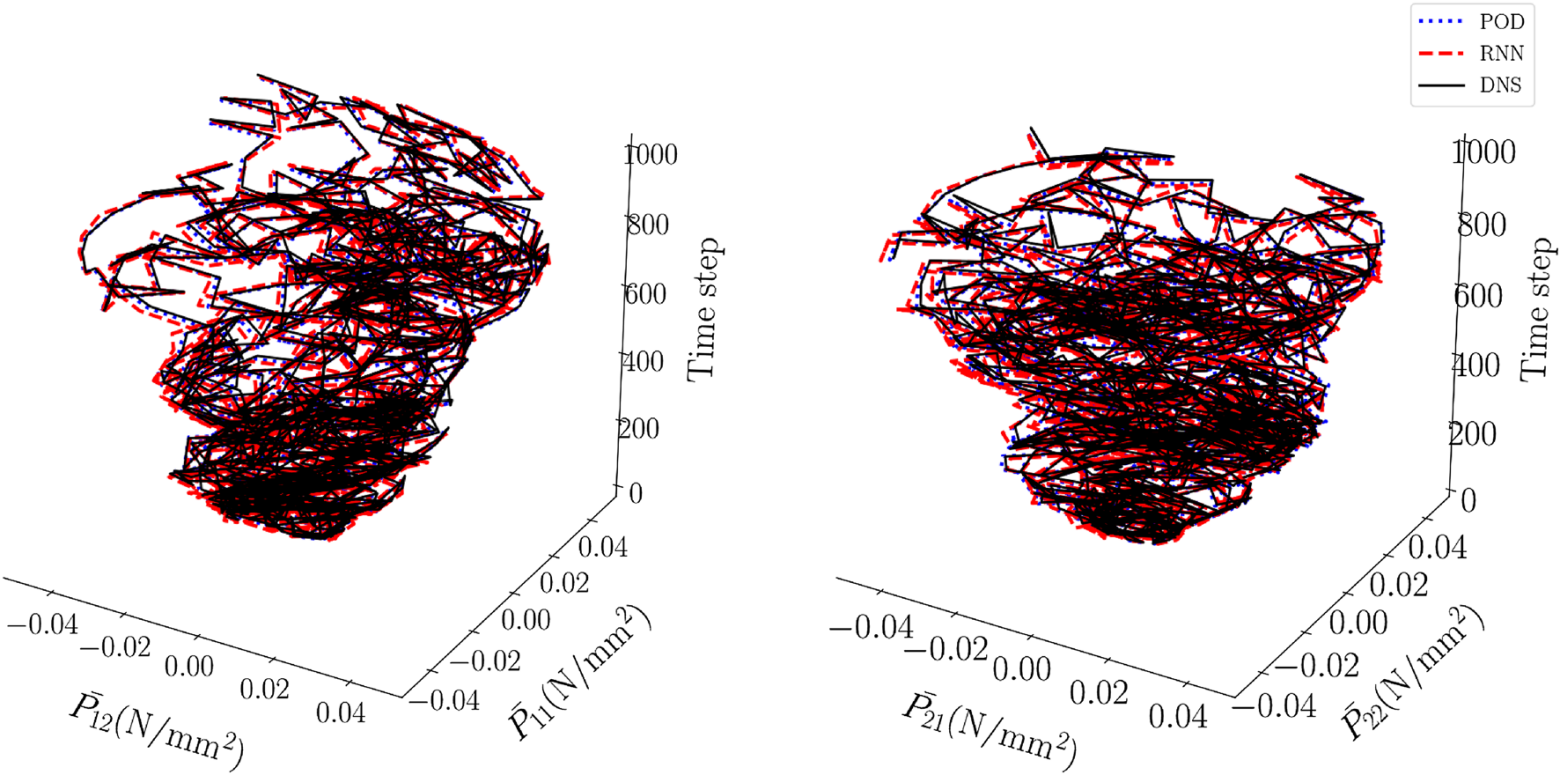
Components of the macroscale $1{st}$ Piola-Kirchhoff stress as functions of the number of increments for a random loading verification simulation.

We continue with results for random loading scenarios. Fig. 12 presents the macroscale, in-plane $1\text {st}$ PK stress components for a random loading verification simulation. Comparing the different predictions with the naked eye demonstrates that the surrogate is fairly accurate for the stringent scenarios of random loading.

**Figure 13:**
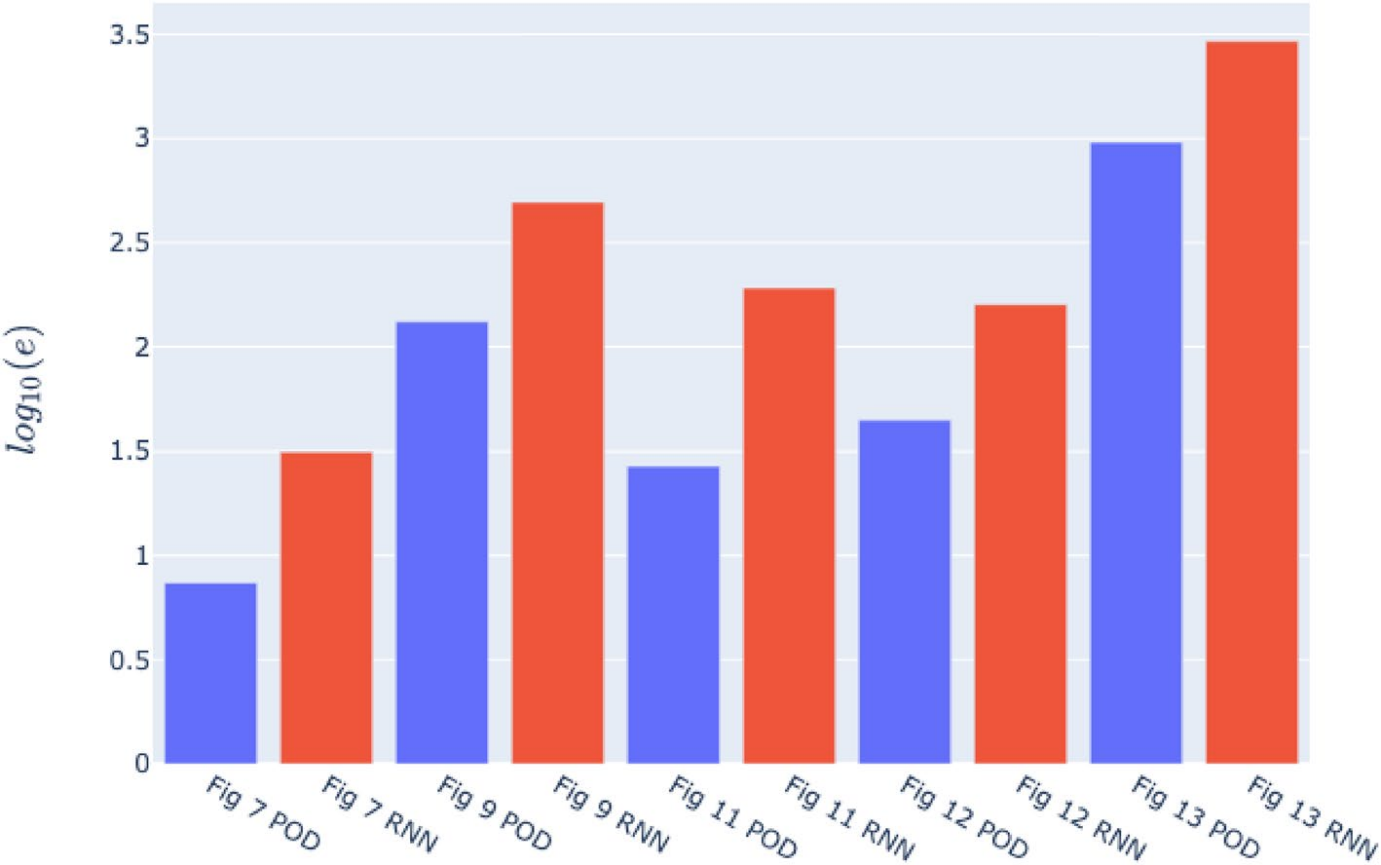
The logarithm of the error of Eq. (31) for the cyclic loading verification simulation (left two bars), the random loading verification simulation (3$\text {th}$ and 4$\text {th}$ bar), the black non-proportional load path in Fig. 14 (5$\text {th}$ and 6$\text {th}$ bar), the yellow non-proportional load path in Fig. 14 (7$\text {th}$ and 8$\text {th}$ bar) and the green non-proportional load path in Fig. 14 (9$\text {th}$ and 10$\text {th}$ bar). The blue bars correspond to the errors of the conventional ROM and the red ones to those of the RNN-POD surrogate.

However, this changes if we introduce an error for the macroscopic stress and compare it to that of the cyclic loading verification simulation. To this purpose, we introduce the following average relative error for the in-plane stress components:

31 $$\begin{aligned} e=\sum _{i}^{n_{incr}}\sum _{jj\in \{11,12,21,22\}}\frac{\arrowvert {\bar{P}}^{i}_{jj}-\tilde{{\bar{P}}}^{i}_{jj}\arrowvert }{\arrowvert {\bar{P}}^{i}_{jj}\arrowvert }, \end{aligned}$$

where the $jj\text {th}$ stress component for the $i\text {th}$ increment computed by the DNS is denoted by ${\bar{P}}^{i}_{jj}$ and the same component resulting from the conventional ROM or the RNN-POD surrogate is denoted by $\tilde{{\bar{P}}}^{i}_{jj}$.

The logarithm of this error is presented in Fig. 13 as the first two bars on the left for the cyclic loading verification simulation (blue: ROM, red: RNN-POD surrogate) and in the subsequent two bars for the random loading verification simulation.

Several observations can be made. First, the conventional ROM for the random case is significantly less accurate than for the cyclic case. In fact, the conventional ROM for the random case is substantially less accurate than the RNN-POD emulator for the cyclic case. The additional increase in error of the RNN-POD emulator is of the same order of magnitude compared to the conventional ROM.

The results discussed so far have focused on cyclic loading and random loading; the scenarios for which the surrogate was trained. At this moment, we can already conclude that the RNN-POD surrogate cannot be used in true multiscale simulations. Although the surrogate is sufficiently accurate for cyclic loading, the resulting smoothness of the resulting macroscopic stress-deformation response is insufficient to drive a multiscale simulation. On top of that, the macroscpic stress-deformation response for random loading is simply inaccurate.

Nevertheless, it is interesting to investigate scenarios for which the RNN is not trained. The reason is that we have assumed that training for random loading and cylic loading will make the RNN capable to emulate any scenario (as previously demonstrated for an RNN that only captures the macroscale stress-deformation relation^9^).

### Non-proportional loading

**Figure 14:**
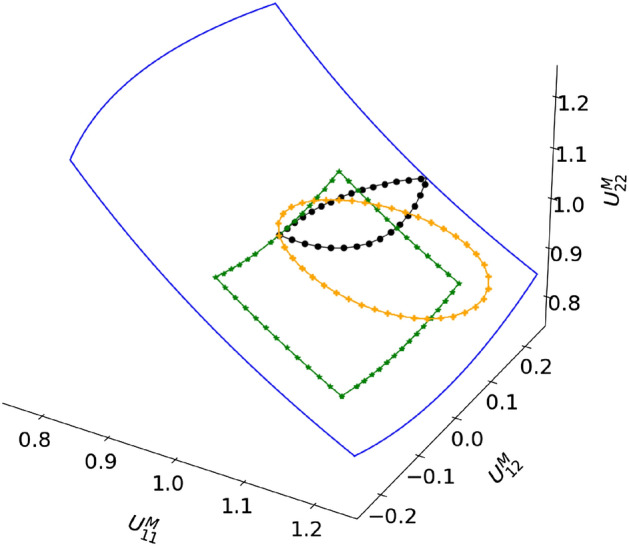
Three non-proportional load paths. The black path is discretized using 1,000 increment, whereas the other two are discretized such that each stepsize is the same as in the random loading simulations used for training.

**Figure 15:**
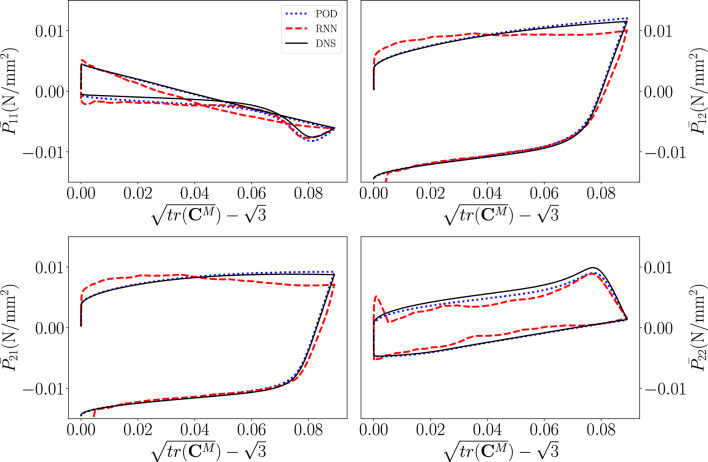
Components of the macroscale $1{st}$ Piola-Kirchhoff stress as functions of the deformation for the non-proportional cyclic load path in black in Fig. 14.

**Figure 16:**
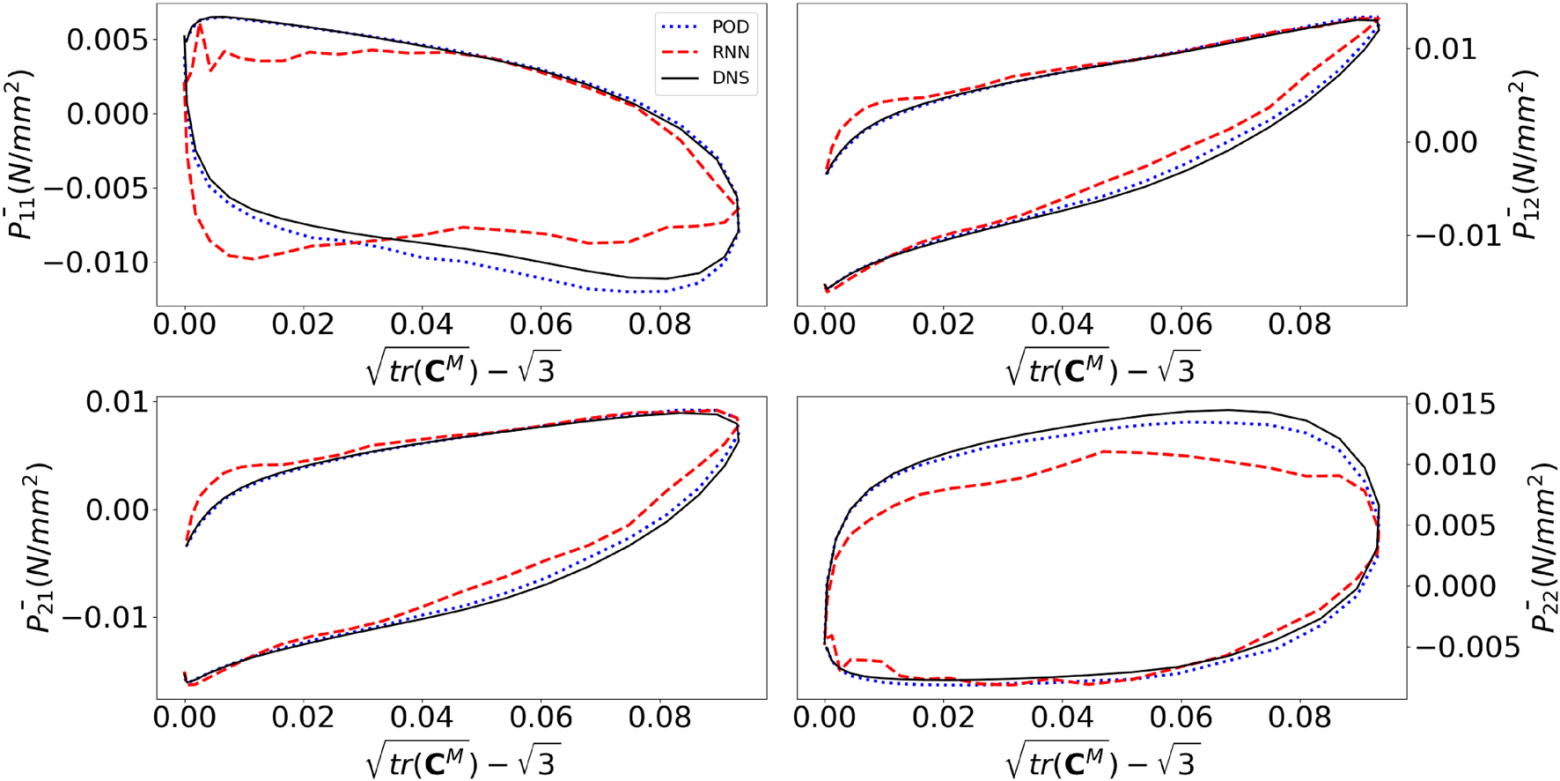
Components of the macroscale $1^{st}$ Piola-Kirchhoff stress as functions of the deformation for the non-proportional cyclic load path in yellow in Fig. 14.

**Figure 17:**
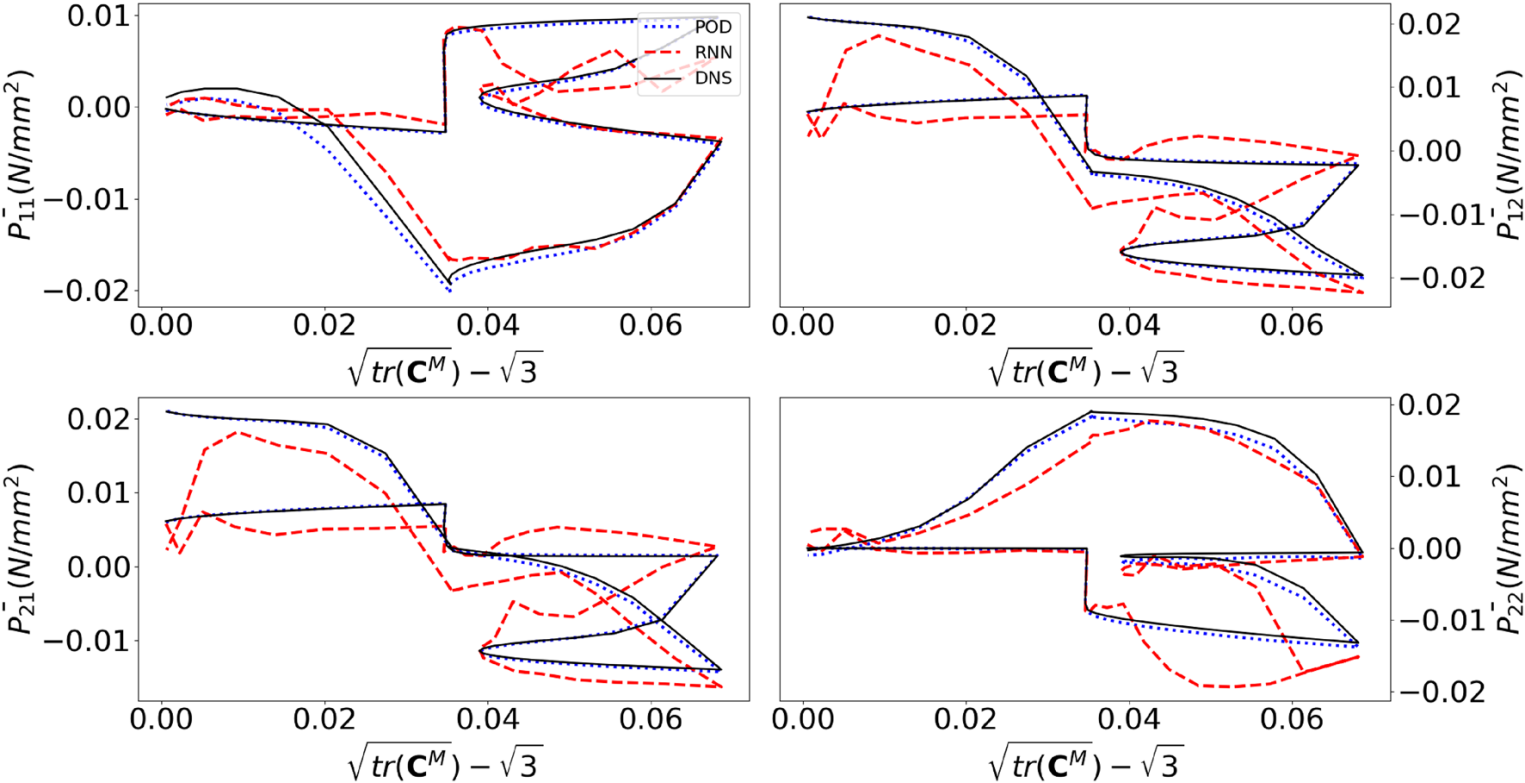
Components of the macroscale $1{st}$ Piola-Kirchhoff stress as functions of the deformation for the non-proportional cyclic load path in green in Fig. 14.

We now focus on the three non-proportional load paths in Fig. 14. The homogenized stress components predicted for these load paths are presented in Figs. 15 for the black load path, Fig. 16 for the yellow load path, and Fig. 17 for the green load path.

We first concentrate on Fig. 15 which presents the $1\text {st}$ PK stress components for the black load path in Fig. 14. It is important to realize that the black load path was discretized using 1,000 increments. Thus, the size of an increment of the black load path, which looks somewhat similar to the cyclic loading simulations used for training, is substantially larger than that of the cyclic loading training simulations, but substantially shorter than that of the random loading training simulations.

The homogenized stress components in Fig. 15 emulated by the RNN-POD framework are clearly substantially worse than those of the investigated cyclic loading verification simulation (Fig. 10). This is also clear from Fig. 13, in which the 5$\text {th}$ (conventional ROM) and 6$\text {th}$ bar (RNN-POD surrogate) present the logarithm of the error of the stress (Eq. (31)). The error is however substantially smaller than for the random loading verification simulation.

One could argue that the emulated stress-deformation curves (Fig. 15) are so poor because the incremental stepsize used to discretize the associated load path (black in Fig. 14) is different from the incremental stepsizes of the cyclic loading training simulations and of the random loading training simulations. For this reason, the yellow and green load paths in Fig. 14 are made such that when discretized, their incremental stepsize is exactly the same as that of the random loading training simulations (i.e. 0.02 in the $U_{11}^M-U_{12}^M-U_{22}^M$ space). However, Figs. 16 and 17 (together with four bars on the right in Fig. 13) demonstrate that the homogenized stress components of the RNN-POD framework for the yellow and green load paths are even worse than those predicted for the black load path. Thus, restricting the stepsize to that of the random loading training simulations (computed with the conventional ROM) has no positive effect on the accuracy of the RNN-POD emulator.

This leads to the conclusion that training the surrogate for random loading and cyclic loading scenarios (two scenarios on opposite sides of the spectrum of possible loading scenarios) does not mean that other loading scenarios will be adequately emulated by the surrogate. As mentioned before, this contrasts previous observations^9^.

### Acceleration

So far we have only discussed accuracy and have ignored speed up. Table 1 summarizes the computational times of the different types of simulations/emulations. Of course, these numbers highly depend on the implementations, hardware, spatial dimensions, type of elements employed in the DNS, number of elements employed in the DNS and more. Consequently, substantial higher speed ups may be realized than reported in Table 1.

Though the data preparation and training the RNN required a total of two weeks of computational time, Table 1 shows that the surrogate is approximately 100 times faster than the DNS and 22 times faster than the conventional MOR for random loading. For cyclic loading on the other hand, the surrogate is only 13 times faster than the DNS, whilst the conventional ROM is hardly faster than the DNS.

**Table:** 

Data preparation	POD	RNN-POD
Cyclic loading	350$\times$1hr	350$\times$1hr
		+
		350$\times$0.75hr
Random loading	1,000$\times$7hr	1,000$\times$7hr
		+
		10,000$\times$1.5hr

**Table 1:** Average computational times for data preparation and the online verification simulations/emulations.

Online stage	DNS	POD	RNN-POD
Cyclic loading	55 min	50 min	4 min
Random loading	7 hr	1.5 hr	4 min

The difference in time savings of the surrogate for cyclic and random loading are because the DNS and conventional MOR require more iterations for random loading than for cyclic loading. The reasons are that (1) the loading paths for cyclic loading are substantially shorter than those for random loading whilst the same number of increments is employed, and (2) the previous plastic state is assumed at the start of each increment of a cyclic loading simulation (i.e. DNS and conventional ROM) in order to increase the speed of the cyclic loading simulations.

## Conclusion

An RNN-POD surrogate model is investigated that emulates the displacement field of each increment of a rate-independent elastoplastic simulation. A recurrent neural network (RNN) in the surrogate is responsible for the emulation. In order to reduce the number of output variables of the RNN, proper-orthogonal-decomposition (POD) is exploited to efficiently reconstruct each displacement field as the sum (of a relatively small number) of orthogonal modes, each multiplied by a single coefficient. Consequently, the RNN only has to emulate the coefficient of each mode (instead of the displacement vector of each finite element node).

We have applied the framework to an elastoplastic RVE. Whereas many neural networks^1,8,9,14^ aim to rapidly emulate the homogenized stress-deformation of an RVE (which drives multiscale simulations), they lose all microstructural information. The RNN-POD surrogate has the potential to both drive multiscale simulations, as well as to recover any microstructural field of interest.

Because the surrogate emulates the displacement field in the entire RVE domain, the non-linear governing equations of the RVE simulations do not need to be constructed, nor solved. Instead, only the new plastic variables must be computed once per increment (together with any other quantity of interest). The time savings are thus substantial and we report a maximum acceleration factor of 100 relative to the direct numerical simulations.

Instead of a conventional feed-forward neural network (FNN), an RNN is used in the surrogate. The reason is that RNNs predict both the primary output of interest, as well as hidden states, which affect the RNN’s next prediction. Thus, the role of hidden states in RNNs imitates the role of plastic variables in elastoplastic finite element simulations.

Although we report acceleration factors of up to 100, the accuracy of the surrogate leaves much to be desired. The reconstruction of the displacement field using the POD modes definitely reduces the accuracy (in terms of both macroscale stress-deformation relation, as well as the recovery of a microstructural field), but the macroscale stress-deformation relations predicted by the conventional ROM seem to be just as smooth as those of the DNS. Hence, we believe that the inaccuracy introduced by the POD modes would not affect the convergence of a multiscale simulation.

On the other hand, the RNN introduces both a substantial inaccuracy in the macroscale stress-deformation curves, as well as a substantial reduction of the smoothness of the macroscale stress-deformation curves. Only the macroscopic stress-deformation curves for the cyclic load case are accurate (but lack smoothness). We want to emphasize that this is in contrast to the results of Wu et al.^9^, in which the same RNN was used to only emulate the macroscale stress-deformation curves. Apparently, an RNN that mimics an elastoplastic constitutive response may not be sufficient to emulate the modal coefficients of an elastoplastic ROM.

In conclusion, we can state that the RNN-POD surrogate, as presented here, cannot emulate the microstructural fields sufficiently accurate to truly *drive* multiscale simulations. Potential avenues to accomplish this aim are:

- A wider variety of load paths and of incremental step sizes of the macroscale deformation may be incorporated in the training data of the RNN.
- Another type of RNN may increase the accuracy. For instance, the RNN-POD framework of Im et al.^26^ uses two long-short-term-memory (LSTM) units in parallel (whereas we have only used a single GRU) Another possible avenue are the RNNs of Liu et al.^15^ and Bhattacharya et al.^16^. In contrast to our GRU and the LSTM of Liu et al.^26^ in which the quantity of current interest and the updated hidden states are one and the same, the output of current interest and the updated hidden states are truly separate in the RNNs of Liu et al.^15^ and Bhattacharya et al.^16^, potentially increasing the accuracy.
- In order to drive multiscale simulations, the sensitivity of the macroscale stress-deformation relation must be sufficiently smooth. The presented results have revealed that the RNN emulation (and not the POD construction of the displacement field in the ROM) is responsible for the lack of smoothness. A potential avenue may therefore be to not only train the RNN to emulate the displacement field itself, but also to train the RNN’s sensitivity according to the sensitivity of the macroscale stress-deformation relation.

Finally, it is worth to mention that the accuracy of our conventional ROM was also not great, although 100 modes were employed. To overcome this problem, one may use non-linear MOR^30,31^. If non-linear MOR is used in combination with the RNN emulation of the model coefficients however, the RNN must not only be able to treat the ‘elastoplasticity’ in its emulation (as in the current contribution), but it must also be able to treat the evolution of the POD modes during the course of a simulation/emulation.

## Data Availability

The corresponding author will send the data upon reasonable request.
